# Effect of Vitamin D Supplementation on Inflammatory Markers in Obese Patients with Acute and Chronic Orthopedic Conditions

**DOI:** 10.3390/nu16213735

**Published:** 2024-10-31

**Authors:** Michał Gawryjołek, Michał Wiciński, Maria Zabrzyńska, Jakub Ohla, Jan Zabrzyński

**Affiliations:** 1 Department of Orthopaedics and Traumatology, Dr L. Blazek Multi-Specialty Hospital, 88-100 Inowroclaw, Poland; 2 Department of Pharmacology and Therapeutics, Faculty of Medicine, Collegium Medicum in Bydgoszcz, Nicolaus Copernicus University, M. Curie 9, 85-090 Bydgoszcz, Poland; 3 Faculty of Medicine, Collegium Medicum in Bydgoszcz, Nicolaus Copernicus University in Torun, 85-067 Bydgoszcz, Poland; maria.zabrzynska@gmail.com; 4 Department of Orthopaedics and Traumatology, Faculty of Medicine, Collegium Medicum in Bydgoszcz, Nicolaus Copernicus University in Torun, 85-092 Bydgoszcz, Poland; jakub.ohla@wp.pl (J.O.); zabrzynski@gmail.com (J.Z.)

**Keywords:** vitamin D, obesity, inflammatory process

## Abstract

Numerous studies have shown that vitamin D may play an important role in modulating the inflammatory process. This study aimed to evaluate the effect of vitamin D supplementation on inflammatory markers in patients with orthopedic disorders and obesity. Thirty-three obese subjects were included in the study and were divided into two groups based on their medical condition: acute orthopedic diseases and chronic orthopedic diseases. Inclusion criteria for the research included age 18–75 years, BMI > 30 kg/m^2^, vitamin D deficiency, and no previous vitamin D supplementation. Samples were collected before and after 3 months of 4000 IU/day vitamin D supplementation. The study used enzyme-linked immunosorbent assay (ELISA) and measured serum levels of markers such as chitinase-3-like protein 1 (YKL-40), interleukin 6 (IL-6), interleukin 17 (IL-17), tumor necrosis factor (TNF-α), and adiponectin. After 3 months of vitamin D supplementation, a statistically significant increase in vitamin D and IL-17 levels was observed in the group with acute orthopedic diseases. Similarly, after supplementation, a statistically significant increase in vitamin D, IL-6 and TNF-α levels was observed in the group with chronic orthopedic diseases. Moreover, after vitamin D supplementation, statistically significantly higher adiponectin levels were observed in the chronic orthopedic group than in the acute orthopedic group. Despite high-dose vitamin D supplementation, inflammatory markers increased in acute and chronic orthopedic conditions. Based on our study, vitamin D does not reduce inflammation in patients with orthopedic conditions and obesity.

## 1. Introduction

According to the World Health Organization, overweight and obesity is a condition in which excessive or abnormal accumulation of body fat increases health risks. Obesity is caused by the abnormal accumulation of body fat, leading to an imbalance between energy intake and energy expenditure [1]. Obesity is believed to be associated with an inflammatory response. Epidemiological studies of people with obesity have shown that elevated levels of inflammatory cytokines accompany obese people. In obese patients, serum often occurs at high levels of inflammatory cytokines, such as tumor necrosis factor (TNF-α) and interleukin families, especially interleukin 6 (IL-6) [2,3].

The adipose tissue itself leads to inflammation. Adipose tissue can secrete adipokines and inflammatory factors. It should not be treated simply as an energy store or metabolic organ but also as an endocrine organ [4,5]. Adipocytokines may mediate inflammation [6,7]. It has also been proven that obesity increases the migration of macrophages to adipose tissue [8]. Increased numbers and activation of immune cells, including macrophages, neutrophils, and helper T cells, characterize obesity-induced inflammation. This produces pro-inflammatory cytokines such as TNF-α, interleukins, and C-reactive proteins (CRP). At the same time, it inhibits anti-inflammatory cells, reduces adiponectin production and predisposes to various cellular stresses, such as endoplasmic reticulum (ER) stress, mitochondrial dysfunction, and oxidative stress [9].

Bone healing processes are linked to acute inflammation and the innate immune system. In response to a disruptive stimulus that threatens the existence or function of the organism, the innate immune system is activated to reestablish a normal state of homeostasis. Circulating and local monocytes and macrophages are activated and respond immediately to the adverse stimuli with a pre-programmed series of non-antigen-specific events. Monocytes/macrophages sense and regulate subsequent biological events to attenuate the adverse stimulus and restore normal local anatomy and physiology [10,11,12,13].

Inflammation associated with osteoarthritis is a pathophysiological phenomenon that involves many cell types and tissues within and outside the joint. Limited inflammation is beneficial and necessary for tissue repair. However, uncontrolled, dysregulated, unremitting inflammation underlies chronic inflammatory conditions [14]. Not only synovitis but also articular cartilage, meniscus, and subchondral bone also participate in inflammatory interactions during joint disease. Over the past two decades, the role of inflammation in osteoarthritis has been well established [15,16,17]. Despite this, many unresolved questions remain regarding its underlying mechanisms.

Vitamin D is a steroid hormone whose active form has multiple biological effects, including regulation of the immune response. The active metabolite can occur in human immune cells such as T cells, B cells, monocytes, and macrophages [18]. The active form of vitamin D, 1,25(OH)2D, participates in immunomodulation via its VDR receptors expressed on human immune cells, especially in chronic inflammatory disorders, decreasing levels of pro-inflammatory cytokines [19]. Vitamin D mainly affects T helper cells, their proliferation, and cytokines production: IL-2, IFNc, TNF-α, IL-3, IL-4, IL-5, IL-10, and IL-17 [3]. The deficiency of vitamin D has been associated with immunomodulation and initiation of many diseases [20].

Holick and Giulietti reported that vitamin D may play an important role in modulating inflammation [21,22]. Normal blood levels of vitamin D are needed for an optimal anti-inflammatory response in humans [23]. In studies on cell cultures, 1α,25(OH)2D3 inhibits the activation of the nuclear factor kappa-light-chain-enhancer of activated B cells (NF-κB) pathway, the activity of TNF-α, and the release of IL-6 in human endothelial cells, which leads to anti-inflammatory effects [24,25].

Other actions have been noted in inflammatory cells. In mouse macrophages, treatment with 1α,25(OH)2D3 inhibited the translocation of NF-κB into the nucleus, which resulted in reduced expression of TNF-α [26]. Human monocytes treated with 1α,25(OH)2D3 suppressed the harmful effects of TNF-α by inhibiting the expression of its receptors [23]. Similar results were reported in human studies where monocytes from patients with type 2 diabetes (T2D) showed significantly higher levels of pro-inflammatory cytokines, including TNF-α, IL-6, and IL-1, compared to monocytes from healthy controls. However, treatment with 1α,25(OH)2D3, mediated by MKP-1 (MAPK-1 phosphatase), inhibited the expression of pro-inflammatory cytokines [21]. Data examining the effect of vitamin D supplementation suggest various beneficial effects of vitamin D, including anti-inflammatory effects.

The Endocrine Society, the National and International Osteoporosis Foundation, and the American Geriatric Society define vitamin D deficiency as the level of 25-hydroxyvitamin (25 OH D) of less than 30 ng/mL. The Endocrine Society recommends a preferred range of 40 to 60 ng/mL.

The aim of this study was to check how vitamin D supplementation could aid in suppressing the inflammatory process caused by either obesity or orthopedic disorders.

We hypothesized that there would be a significant decrease in pro-inflammatory cytokines after vitamin D supplementation in our population of obese patients with acute and chronic orthopedic disorders.

## 2. Material and Methods

### 2.1. Bioethics

The study was conducted following the Basic and Clinical Pharmacology and Toxicology Policy for Experimental and Clinical studies. (approval number: KB 465/2022, approval date: 27 September 2022). The study was performed by the Declaration of Helsinki for experiments involving humans after receiving permission from the Bioethics Committee of Collegium Medicum in Bydgoszcz, Nicolaus Copernicus University in Toruń. All patients were volunteers and gave informed consent.

### 2.2. Study Cohort

The study included 33 patients recruited from the Department of Orthopedic Surgery. The patients were selected for the study based on the following inclusion criteria: age 18–75, BMI > 30 kg/m^2^, vitamin D insufficient < 30 ng/mL. The exclusion criteria were pregnancy, taking anticoagulants regularly, cancer, dialysis, and liver disease. None of the patients had previously supplemented with vitamin D. The subjects included in the study were divided into two groups: acute orthopedic diseases and chronic orthopedic diseases. The acute orthopedic diseases group included subjects with broken bones, sprained joints, and damage to the meniscus of the knee joint. The chronic orthopedic diseases group included patients with osteoarthritis of the knee and hip joint. The tests were carried out from September 2022 to May 2023, eliminating the impact of UV-B radiation. None of the patients changed their diet habits during the follow-up. In addition, data were collected on the age, weight, and height of the subjects.

### 2.3. Samples Collection

A peripheral vein blood sample was collected from each participant twice—before and after the 3 months of vitamin D supplementation. The serum from these samples was separated and immediately transported to the Department of Pharmacology and Therapeutics, Collegium Medicum in Bydgoszcz.

### 2.4. Outcome Evaluation

Serum and plasma were prepared immediately according to standard procedure. The material was frozen at −20 °C, and samples were then transported on dry ice to the Department of Pharmacology and Therapeutics, where they were stored at −70 °C until analysis. Serum protein markers such as YKL-40, IL-17, IL-6, TNF-α, and adiponectin were measured in all patients using an enzyme-linked immunosorbent assay (ELISA) (Sunredbio (SRB) Technology, Shanghai, China; DRG Instruments GmbH, Marburg, Germany; Mediagnost GmbH, Reutlingen, Germany). According to the manufacturer’s instructions, analyses were performed on an EPOCH microplate spectrophotometer (BioTech, Santa Clara, CA, USA).

### 2.5. Measurement of Vitamin D

Serum vitamin D concentration was measured in the hospital laboratory operating at the orthopedic clinic. A sample was taken from the patient’s venous blood at the first visit. A second sample was taken after 3 months of vitamin D supplementation at a dose of 4000 IU per day, orally, in the morning.

### 2.6. Statistical Analysis

Results for each group (‘before supplementation-1’ and ‘after supplementation-2’) were expressed as mean ± standard error of the mean (SEM). Variables were tested for normality of distribution using the Shapiro–Wilk test. Within each group, markers and vitamin D concentrations were compared before and after supplementation using the Wilcoxon test or the *t*-test for dependent samples. The Mann–Whitney U test was used to compare independent groups (acute orthopedic diseases vs. chronic orthopedic diseases). Moreover, using the *t*-test for independent samples or the Mann–Whitney test, inflammatory markers and vitamin D concentrations were also compared by sex in the group with acute orthopedic conditions. Spearman’s rank correlation was used to assess the relationship between marker concentrations, age and BMI of the subjects in each group. Additionally, the correlation between sex and the concentration of inflammatory markers in the group with acute orthopedic conditions was checked. All statistical analyses were performed using Statistica 13.3 software. A *p*-value < 0.05 was considered statistically significant.

## 3. Results

**Figure 1:**
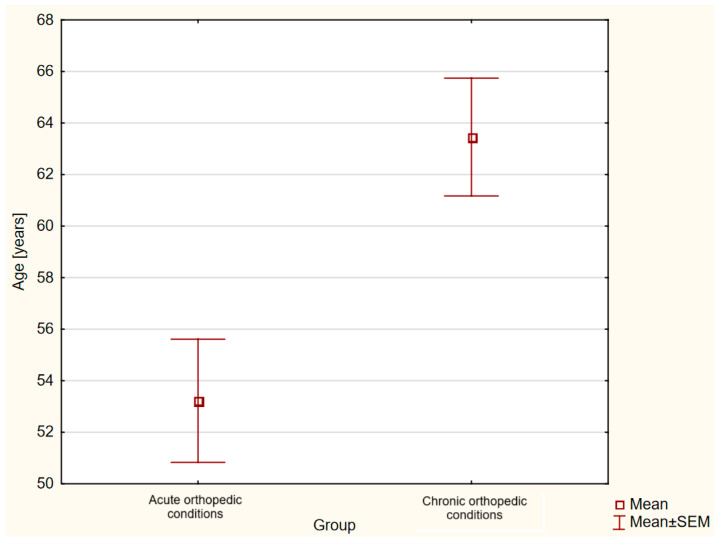
Comparison of mean age of patients in the group with acute orthopedic conditions and chronic orthopedic conditions (*p* = 0.002).

The 33 obese patients included in the study were divided into two groups—acute orthopedic diseases and chronic orthopedic diseases. There were 18 subjects (34% males and 66% females) in the acute disease group and 15 subjects (7% males and 93% females) in the chronic disease group. The mean age in the acute group was 53.22 years (±2.4 SEM), and in the chronic group, 63.47 years (±2.29 SEM). Participants in the chronic group were statistically significantly older (*p* = 0.002) compared to those in the other group (Figure 1). The mean BMI for both groups—acute and chronic—was 33.61 kg/m^2^ (±0.53 SEM) vs. 33.67 kg/m^2^ (±0.61 SEM), respectively, and was not statistically significantly different.

Spearman’s rank correlation was performed to check whether the inflammatory markers and vitamin D selected for the study had significant correlations with the BMI values and age of the patients in each study group. In both the acute orthopedic group (Table 1) and the chronic orthopedic group (Table 2), there were no statistically significant correlations between marker concentrations—TNF-α, adiponectin, IL-6, IL-17, YKL-40, and vitamin D—and age or BMI.

In the group with acute orthopedic diseases, markers and vitamin D concentrations were compared before and after a 3-month supplementation with vitamin D at a dose of 4000 IU/day. Table 3 shows the mean ± SEM for both measurements in this study group.

After 3 months of vitamin D supplementation, a statistically significant increase in serum levels of this vitamin (19.44 ± 1.4 vs. 33.83 ± 3.12 ng/mL; *p* < 0.001) and an increase in IL-17 levels (361.76 ± 46.89 vs. 401.1 ± 42.81 pg/mL; *p* = 0.019) were observed in this group. Concentrations of other markers did not change in a statistically significant manner.

In the group with chronic orthopedic diseases, markers and vitamin D concentrations were compared before supplementation and after a 3-month supplementation with vitamin D at a dose of 4000 IU/day. Table 4 shows the mean ± SEM for both measurements in this study group.

After 3 months of vitamin D supplementation, a statistically significant increase in serum levels of this vitamin (20.39 ± 1.66 vs. 35.22 ± 2.46 ng/mL; *p* < 0.001) and an increase in IL-6 (8.33 ± 0.86 vs. 12.08 ± 1.67 pg/mL; *p* = 0.023) and TNF-α (7.85 ± 1.72 vs. 9.97 ± 1.64 pg/mL; *p* = 0.023) levels were observed in this group. Concentrations of other markers did not change in a statistically significant manner.

Serum markers and vitamin D concentrations were also compared between the two groups, acute and chronic, before supplementation (Table 5) and then after 3 months of supplementation (Table 6).

Before supplementation, there were no statistically significant differences in markers and vitamin D levels between the acute orthopedic group and the chronic orthopedic group.

After 3 months of vitamin D supplementation, statistically significantly higher (*p* = 0.034) adiponectin levels were noted in the chronic orthopedic diseases group (48.47 ± 7.31 ng/mL) than in the acute orthopedic diseases group (29.42 ± 3.76 ng/mL). For the other examined proteins, there were no statistically significant differences.

**Figure 2:**
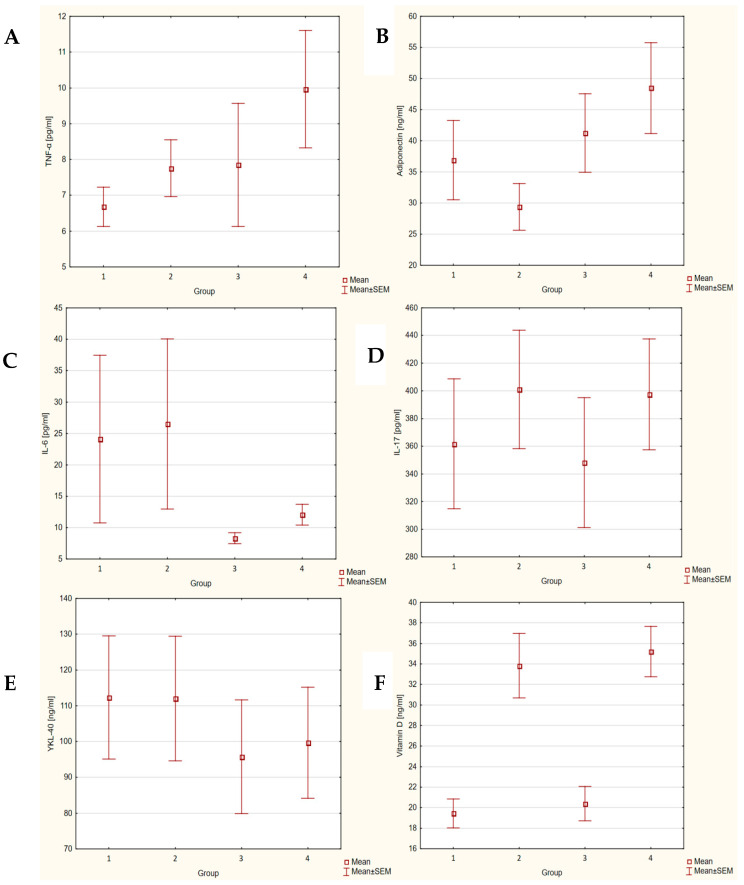
Serum concentrations of markers ((**A**) TNF-α; (**B**) adiponectin; (**C**) IL-6; (**D**) IL-17; (**E**) YKL-40; (**F**) vitamin D) in the group with acute orthopedic diseases (1—before supplementation; 2—after supplementation) and chronic orthopedic diseases (3—before supplementation; 4—after supplementation).

The box plots show a comparison of the concentrations of all markers and vitamin D in each of the study groups before and after supplementation (Figure 2).

**Figure 3:**
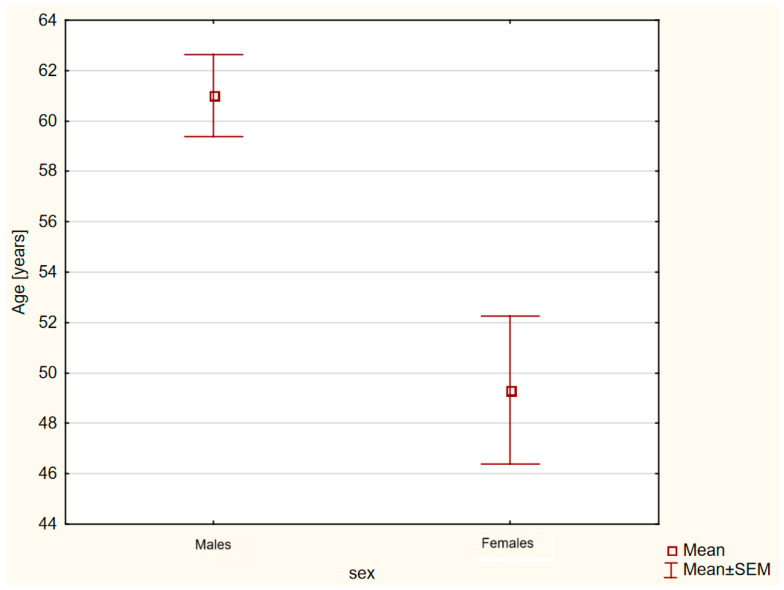
Age comparison of subjects in the group with acute orthopedic conditions according to sex (*p* = 0.017).

Due to the large sex disproportion in the group with acute orthopedic conditions, additional statistical analyses were performed. There were no statistically significant differences between females and males in the group with acute orthopedic conditions in terms of BMI (33.83 ± 0.72 vs. 33.17 ± 0.75 kg/m^2^; *p* = 0.57). On the other hand, it was noted that males in this group were statistically significantly older (*p* = 0.017) than females, as shown in Figure 3.

Serum markers and vitamin D concentrations were also compared by sex—females vs. males—before supplementation (Table 7) and after 3 months of vitamin D supplementation (Table 8) in the group with acute orthopedic conditions.

There were no statistically significant differences in TNF-α, adiponectin, IL-6 and vitamin D levels between women and men in the acute orthopedic group before supplementation. In contrast, statistically significant higher levels of IL-17 were noted in females than in males (433.62 ± 60.07 vs. 218.06 ± 18.09 pg/mL; *p* = 0.025). Similarly, higher concentrations in females than in males were obtained for YKL-40 (137.48 ± 22.4 vs. 62.12 ± 7.17 ng/mL; *p* = 0.034).

Furthermore, no statistically significant differences were found in the levels of TNF-α, adiponectin, IL-6 and vitamin D between females and males in the group with acute orthopedic syndrome after 3 months of vitamin D supplementation. However, statistically significantly higher concentrations of IL-17 (472.05 ± 50.36 vs. 259.19 ± 38.63 pg/mL; *p* = 0.014) and YKL-40 (139.64 ± 21.82 vs. 56.79 ± 8.45 ng/mL; *p* = 0.019) were again observed in females than in males.

Spearman’s rank correlation (Table 9) did not show any statistically significant correlations between sex and the concentration of inflammatory markers and vitamin D in the group with acute orthopedic diseases.

## 4. Discussion

This research aimed to check the levels of pro-inflammatory cytokines in obese patients before and after vitamin D supplementation. The obese patients included in the study were divided into two groups—those with acute orthopedic diseases and those with chronic orthopedic diseases. In both groups, after 3 months of vitamin D supplementation at a dose of 4000 IU/day, serum vitamin D levels increased statistically significantly. IL-17 levels increased in the group with acute conditions. In contrast, an increase in IL-6 levels, as well as TNF-α levels, was observed in the group with chronic conditions. Interestingly, after 3 months of vitamin D supplementation, the chronic conditions group had significantly higher adiponectin levels than the acute conditions group. In addition, patients in the group with chronic orthopedic conditions were statistically significantly older than patients with acute orthopedic conditions. Regardless of the type of condition and obesity, age may be a significant factor in increasing inflammation in the body. In contrast, interestingly, despite the higher age of the male patients in the acute orthopedic group, females had higher levels of IL-17 and YKL-40—both before and after vitamin D supplementation. Therefore, it seems that patients’ inflammation may also be influenced by sex. Nevertheless, regarding our study, there were no significant correlations between age and sex and concentrations of inflammatory markers and vitamin D.

The inverse association between vitamin D deficiency and obesity is widely recognized. While lean and obese patients may have similar levels of vitamin D, vitamin management is disturbed. It results from a larger body volume of obesity and accumulates mainly in enhanced compartments: serum, muscle, liver, and adipose tissue, leaving only a small concentration in the circulation [27]. Additionally, obese patients have a lower level of the active form of vitamin D due to a lower concentration of enzymes responsible for its formation [28].

Obesity leads to the remodeling of adipose tissue, changing either the structure or cellular composition. While adipose tissue is also known as an endocrine organ, obesity enhances the production of adipokines, cellular signaling proteins, which have the pro-inflammatory effect, and, what is more, decreased anti-inflammatory adipokines as well [3,29]. High levels of adipokines, inflammatory modulators in obesity, are responsible for the secretion of pro-inflammatory cytokines, such as IFNγ, TNFα, IL-1β, IL-6, and IL-12 [30,31,32,33]. Moreover, anti-inflammatory adipokines are decreased, omentin, isthmin 1, nesfatin 1, and particularly adiponectin, which also decrease the level of TNF-α—as one of the pro-inflammatory effects [34].

Some studies have indicated that vitamin D levels are directly associated with adiponectin and that this association varies across body mass index categories, becoming stronger with increasing indicators [35,36,37,38]. Mousa et al. [39] noted that vitamin D may increase adiponectin concentrations in overweight, obese and vitamin D-deficient adults. Different results were obtained by Gannagé-Yared et al. and Liu et al. in their research; after three months of use of vitamin D supplementation, serum adiponectin levels did not statistically change [36,40]. In our study, vitamin D supplementation did not influence adiponectin, but the level of vitamin D increased significantly. However, the level of TNF-α increased, which is regulated by adiponectin.

On the other hand, pro-inflammatory adipokines also triggered increased secretion of pro-inflammatory cytokines, apart from TNF- α and IL-6, as well. This may confirm that the concentration of adiponectin was too low to properly reduce the secretion of TNF-α, and the levels of pro-inflammatory adipokines were too high, which may indicate no effect of vitamin D supplementation. Wamberg et al. reported that incubation with 1,25(OH)2D decreased the expression of IL-6 in human adipose tissue [41]. Zhang et al. observed that vitamin D inhibits IL-6 and TNF-α production by human monocytes [23]. On the other hand, Jamka et al. noted that vitamin D supplementation did not influence TNF-α and IL-6 concentrations [42]. Vitamin D also influences a third group of T-cells known as Th 17 cells, which reduces the production of IL-17 [43]. However, in our study, the vitamin D supplementation had no effect; quite the opposite, it caused an increase in IL-17 concentrations.

Our findings differ in the study group among patients with acute and chronic disorders, despite the growth of the serum level of vitamin D. Patients with acute disorders have significantly higher levels of IL-17. McGeachy et al. suggested that the IL-17 cytokine family is relatively poorly understood, apart from IL-17A, and increased IL-17 in obesity may reflect an attempt by the immune system to correct the pathologic nature of the increased fat tissue and the associated metabolic stress [44]. Jorde et al. measured the pro-inflammatory cytokines level, e.g., IL-17 in overweight patients, after one-year intervention with 40,000 IU vitamin D per week, 20,000 IU vitamin D per week, or placebo, and they were not able to demonstrate with certainty any significant relationship between serum 25(OH)D levels and some cytokines and markers of inflammation [45].

Interleukin 6 is mainly produced and significantly increases in infections and tissue injuries, supporting acute phase responses but also in autoimmune diseases [46]. On the other hand, patients with chronic disorders mainly have higher levels of TNF-α and also IL-6. In the group of our chronic patients prevail osteoarthritis and meniscal tears, which can result in higher levels of TNF- α and IL-6. Osteoarthritis has an impact on pro-inflammatory adipokinase. The level and activity of leptin in articular cartilage is increased, which stimulates the production of TNF- α and IL-6 [47,48,49]. Beilfuss et al. showed that a one-year intervention with vitamin D decreased serum IL-6 levels but did not affect TNF-α levels [50]. Additionally, Wiciński et al. presented that three months of vitamin D 2000 IU therapy did not induce any statistically significant changes in serum levels of IL-6 [51]. In our study, vitamin D probably did not affect serum levels of the above-mentioned cytokines; quite the opposite, the levels statistically significantly increased.

Overall, the multiple orthopedic diseases in our population itself influenced our results. Our population comprised 33 obese patients with the implication of ongoing inflammation. Additionally, acute and chronic orthopedic conditions strengthen the inflammation process. Thus, the dose of vitamin D in our study may not be enough to achieve the decrease of pro-inflammatory markers’ levels. Karonova et al. presented a significant decrease in IL-6 only in patients who received high-dose vitamin D therapy (40,000 IU per week); no changes were detected in the 5000 IU per week group [52].

Several limitations were noted in this study. The main limitations of this study were the modest number of included patients and a short period of vitamin D supplementation. Additionally, the patients were diagnosed with various orthopedic conditions. This could potentially affect the various levels of cytokines in our study and cause greater differences in our results. A more homogeneous population in terms of orthopedic conditions may result in more reliable outcomes. It is worth mentioning that inflammation is also affected by age and sex, which should be taken into account when evaluating the potential anti-inflammatory effect of vitamin D. Due to limited physical activity because of orthopedic conditions, patients tended to lead a sedentary lifestyle, which was not conducive to weight loss and may have contributed to inflammation. Furthermore, we could not obtain reliable information about the consistency in supplementing vitamin D. However, statistically significant increased vitamin D serum proves otherwise.

## 5. Conclusions

Our study findings indicate that after vitamin D supplementation, the levels of pro-inflammatory cytokines increased. However, besides obesity, patients were diagnosed with various orthopedic conditions. This could potentially affect the various levels of cytokines and cause their growth, which may indicate that the vitamin D dose was too low to affect the inflammation process. On the other hand, comparing the results between acute and chronic conditions groups, the only statistically significant change was in the concentration of adiponectin, which speaks of no difference between the effect of supplementation with vitamin D comparing patients with acute and chronic disorders. Based on our study, vitamin D does not reduce inflammation in patients with orthopedic conditions and obesity.

## Figures and Tables

**Table 1:** Spearman’s rank correlation between inflammatory markers, vitamin D, BMI and age in a group with acute orthopedic conditions.

		Acute Orthopedic Conditions			
		*N*	R Spearman	t (*N* − 2)	*p*-Value
BMI	TNF-α	18	0.26	1.08	>0.05
	Adiponectin	18	−0.04	−0.15	
	IL-6	18	0.08	0.31	
	IL-17	18	−0.22	−0.92	
	YKL-40	18	−0.17	−0.68	
	Vitamin D	18	0.05	0.18	
Age	TNF-α	18	0.18	0.73	>0.05
	Adiponectin	18	−0.09	−0.37	
	IL-6	18	0.36	1.53	
	IL-17	18	−0.30	−1.24	
	YKL-40	18	−0.46	−2.08	
	Vitamin D	18	−0.47	−2.13	

**Table 2:** Spearman’s rank correlation between inflammatory markers, vitamin D, BMI, and age in a group with chronic orthopedic conditions.

		Chronic Orthopedic Conditions			
		*N*	R Spearman	t (*N* − 2)	*p*-Value
BMI	TNF-α	15	−0.48	−1.98	>0.05
	Adiponectin	15	0.00	0.00	
	IL-6	15	−0.42	−1.69	
	IL-17	15	0.48	1.99	
	YKL-40	15	0.49	2.01	
	Vitamin D	15	−0.11	−0.39	
Age	TNF-α	15	0.26	0.96	>0.05
	Adiponectin	15	−0.51	−2.15	
	IL-6	15	−0.17	−0.63	
	IL-17	15	−0.18	−0.66	
	YKL-40	15	−0.07	−0.26	
	Vitamin D	15	−0.11	−0.41	

**Table 3:** Mean and standard error of the mean (SEM) of markers and vitamin D serum concentrations in the group with acute orthopedic conditions (1—before supplementation; 2—after 3-month vitamin D supplementation).

	Acute Orthopedic Conditions		
Protein	Mean ± SEM (1)	Mean ± SEM (2)	*p*-Value
TNF-α [pg/mL]	6.68 ± 0.55	7.76 ± 0.79	0.285
Adiponectin [ng/mL]	36.9 ± 6.39	29.42 ± 3.76	0.099
IL-6 [pg/mL]	24.11 ± 13.36	26.5 ± 13.54	0.052
IL-17 [pg/mL]	361.76 ± 46.89	401.1 ± 42.81	0.019
YKL-40 [ng/mL]	112.36 ± 17.2	112.02 ± 17.38	0.937
Vitamin D [ng/mL]	19.44 ± 1.4	33.83 ± 3.12	<0.001

**Table 4:** Mean and standard error of the mean (SEM) of markers and vitamin D serum concentrations in the group with chronic orthopedic conditions (1—before supplementation; 2—after 3-month vitamin D supplementation).

	Chronic Orthopedic Conditions		
Protein	Mean ± SEM (1)	Mean ± SEM (2)	*p*-Value
TNF-α [pg/mL]	7.85 ± 1.72	9.97 ± 1.64	0.023
Adiponectin [ng/mL]	41.27 ± 6.30	48.47 ± 7.31	0.307
IL-6 [pg/mL]	8.33 ± 0.86	12.08 ± 1.67	0.023
IL-17 [pg/mL]	348.2 ± 47.06	397.55 ± 39.99	0.117
YKL-40 [ng/mL]	95.71 ± 15.88	99.69 ± 15.51	0.182
Vitamin D [ng/mL]	20.39 ± 1.66	35.22 ± 2.46	<0.001

**Table 5:** Comparison of markers and vitamin D serum concentrations before supplementation (1) in the group with acute orthopedic conditions and the group with chronic orthopedic conditions.

	Acute Orthopedic Conditions	Chronic Orthopedic Conditions	
Protein	Mean ± SEM (1)	Mean ± SEM (1)	*p*-Value
TNF-α [pg/mL]	6.68 ± 0.55	7.85 ± 1.72	0.871
Adiponectin [ng/mL]	36.9 ± 6.39	41.27 ± 6.30	0.504
IL-6 [pg/mL]	24.11 ± 13.36	8.33 ± 0.86	0.255
IL-17 [pg/mL]	361.76 ± 46.89	348.2 ± 47.06	0.898
YKL-40 [ng/mL]	112.36 ± 17.2	95.71 ± 15.88	0.869
Vitamin D [ng/mL]	19.44 ± 1.4	20.39 ± 1.66	0.539

**Table 6:** Comparison of markers and vitamin D serum concentrations after supplementation (2) in the group with acute orthopedic conditions and the group with chronic orthopedic conditions.

	Acute Orthopedic Conditions	Chronic Orthopedic Conditions	
Protein	Mean ± SEM (2)	Mean ± SEM (2)	*p*-Value
TNF-α [pg/mL]	7.76 ± 0.79	9.97 ± 1.64	0.247
Adiponectin [ng/mL]	29.42 ± 3.76	48.47 ± 7.31	0.034
IL-6 [pg/mL]	26.5 ± 13.54	12.08 ± 1.67	0.481
IL-17 [pg/mL]	401.1 ± 42.81	397.55 ± 39.99	0.956
YKL-40 [ng/mL]	112.02 ± 17.38	99.69 ± 15.51	0.985
Vitamin D [ng/mL]	33.83 ± 3.12	35.22 ± 2.46	0.842

**Table 7:** Comparison of markers and vitamin D serum concentrations before supplementation (1) in the group with acute orthopedic conditions according to sex.

	Females (*N* = 12)	Males (*N* = 6)	
Protein	Mean ± SEM (1)	Mean ± SEM (1)	*p*-Value
TNF-α [pg/mL]	6.87 ± 0.63	6.29 ± 1.15	0.639
Adiponectin [ng/mL]	43.75 ± 7.8	23.18 ± 9.59	0.133
IL-6 [pg/mL]	30.26 ± 20.06	11.8 ± 2.32	0.531
IL-17 [pg/mL]	433.62 ± 60.07	218.06 ± 18.09	0.025
YKL-40 [ng/mL]	137.48 ± 22.4	62.12 ± 7.17	0.034
Vitamin D [ng/mL]	19.99 ± 1.75	18.33 ± 2.49	0.59

**Table 8:** Comparison of markers and vitamin D serum concentrations after supplementation (2) in the group with acute orthopedic conditions according to sex.

	Females (*N* = 12)	Males (*N* = 6)	
Protein	Mean ± SEM (2)	Mean ± SEM (2)	*p*-Value
TNF-α [pg/mL]	7.16 ± 0.98	8.96 ± 1.33	0.301
Adiponectin [ng/mL]	29.82 ± 4.61	28.61 ± 7.09	0.884
IL-6 [pg/mL]	33.73 ± 20.26	12.04 ± 1.59	0.467
IL-17 [pg/mL]	472.05 ± 50.36	259.19 ± 38.63	0.014
YKL-40 [ng/mL]	139.64 ± 21.82	56.79 ± 8.45	0.019
Vitamin D [ng/mL]	33.82 ± 3.87	33.85 ± 5.79	0.99

**Table 9:** Spearman’s rank correlation between inflammatory markers, vitamin D, and sex in a group with acute orthopedic conditions.

		Acute Orthopedic Conditions			
		*N*	R Spearman	t (*N* − 2)	*p*-Value
Sex	TNF-α	18	0.09	0.36	>0.05
	Adiponectin	18	0.43	1.91	
	IL-6	18	−0.20	−0.84	
	IL-17	18	−0.44	−1.96	
	YKL-40	18	0.35	1.48	
	Vitamin D	18	0.09	0.36	

## Data Availability

The original contributions presented in the study are included in the article, further inquiries can be directed to the corresponding author.
